# Correlation between symptoms of depression, attitude, and self-care in elderly with type 2 diabetes

**DOI:** 10.1590/0034-7167-2022-0741

**Published:** 2023-07-10

**Authors:** Maria Cristina Lins Oliveira Frazão, Lia Raquel de Carvalho Viana, Gerlania Rodrigues Salviano Ferreira, Cláudia Jeane Lopes Pimenta, Cleane Rosa Ribeiro da Silva, Kaisy Martins de Albuquerque Madruga, Patrícia Serpa de Souza Batista, Kátia Neyla de Freitas Macedo Costa

**Affiliations:** I Universidade Federal da Paraíba. João Pessoa, Paraíba, Brazil; II Universidade Federal De Campina Grande. Campina Grande, Paraíba, Brazil

**Keywords:** Diabetes Mellitus, Depression, Attitude Towards Health, Self-Care, Elderly Health, Diabetes Mellitus, Depresión, Actitud Frente a la Salud, Autocuidado, Salud del Anciano, Diabetes Mellitus Mellitus, Depressão, Atitude Frente à, Saúde, Autocuidado, Saúde do Idoso

## Abstract

**Objectives:**

to correlate depressive symptoms, attitude, and self-care of elderly people with type 2 diabetes.

**Methods:**

study developed with 144 elderly people with diabetes; carried out in Family Health Units. A semi-structured instrument was used to obtain data on the sociodemographic profile; the Geriatric Depression Scale (15 items), the *Questionário de Atitudes Psicológicas do Diabetes* [Psychological Attitudes of Diabetes Questionnaire], and the Diabetes Self-Care Activities Questionnaire (DSCA) were also used.

**Results:**

it was identified that 24.3% of the participants presented depressive symptoms, and 93.8% presented negative attitudes of coping. A greater adherence to self-care activities related to the practice of medication was observed. In the correlation between the scales, a negative and inversely proportional correspondence was noted between depressive symptomatology and physical activity (p=0.010) and foot care (p=0.006), likewise between attitude and foot care (p=0.009).

**Conclusions:**

self-care in elderly people with diabetes *mellitus* is influenced by depressive symptoms and negative coping attitudes.

## RESUMO

**Objetivos:**

correlacionar sintomas depressivos, atitude e autocuidado de pessoas idosas com diabetes tipo 2.

**Métodos:**

estudo desenvolvido com 144 pessoas idosas com diabetes; realizado em Unidades de Saúde da Família. Utilizaram-se instrumento semiestruturado para obter os dados referentes ao perfil sociodemográfico, a Escala de Depressão Geriátrica (15 itens), o Questionário de Atitudes Psicológicas do Diabetes e o de Atividades de Autocuidado com o Diabetes.

**Resultados:**

identificou-se que 24,3% dos participantes apresentaram sintomatologia depressiva; e 93,8%, atitudes negativas de enfrentamento. Observou-se uma maior adesão às atividades de autocuidado relacionadas à prática medicamentosa. Na correlação entre as escalas, foi percebida uma correspondência negativa e inversamente proporcional entre sintomatologia depressiva e atividade física (p=0,010) e cuidado com os pés (p=0,006), do mesmo modo entre atitude e cuidado com os pés (p=0,009).

**Conclusões:**

o autocuidado em pessoas idosas com diabetes *mellitus* sofre influência dos sintomas depressivos e da atitude negativa no enfrentamento.

## RESUMEN

**Objetivos:**

correlacionar síntomas depresivos, actitud y autocuidado de personas ancianas con diabetes tipo 2.

**Métodos:**

estudio desarrollado con 144 ancianos con diabetes; realizado en Unidades de Salud de la Familia. Utilizados instrumento semiestructurado para obtener los datos referentes al perfil sociodemográfico, Escala de Depresión Geriátrica (15 ítems), Encuesta de Actitudes Psicológicas del Diabetes y de Actividades de Autocuidado con el Diabetes.

**Resultados:**

identificado que 24,3% de los participantes presentaron sintomatología depresiva; y 93,8%, actitudes negativas de enfrentamiento. Observado una mayor adhesión a las actividades de autocuidado relacionadas a la práctica medicamentosa. En la correlación entre las escalas, fue percibida una correspondencia negativa e inversamente proporcional entre sintomatología depresiva y actividad física (p=0,010) y cuidado con los pies (p=0,006), igualmente entre actitud y cuidado con los pies (p=0,009).

**Conclusiones:**

el autocuidado en personas ancianas con diabetes *mellitus* sufre influencia de los síntomas depresivos y de la actitud negativa en el enfrentamiento.

## INTRODUCTION

Chronic noncommunicable diseases occupy a prominent position on the world stage since they cause approximately 38 million deaths among the elderly every year^(1)^. Among these diseases, diabetes mellitus has shown a high and worrisome growth. It is estimated that it may reach 471 million people worldwide in the next decades, with 80% of this increase occurring in lowand middle-income countries^(2-3)^.

In Brazil, almost 14 million people live with diabetes, placing it among the four countries with the highest prevalence, along with India, China, and the United States^(3)^. Studies have highlighted the significant increase in cases after the age of 60, reaching 20% of the population between 65 and 74 years of age, a percentage that corresponds to 3.5 million people^(2-3)^.

Diabetes is a severe clinical syndrome that develops through a heterogeneous metabolic disorder characterized by hyperglycemia resulting from unsatisfactory insulin action^(4)^. Its manifestation interferes with the physical, psychological, and emotional condition and requires adaptation to a new lifestyle to prevent complications^(5-6)^.

Living with diabetes can cause depressive symptoms, such as feelings of hopelessness, fear, and sadness, which may hinder the coping with the disease and impair its treatment. Researchers identified difficulty in following the recommended diet and daily insulin application in elderly people diagnosed with diabetes and depressive symptoms^(7-8)^.

The prevalence of depressive symptoms in patients with this pathology can reach a percentage higher than 40%^(9)^. This symptomatology can cause a negative attitude towards the disease, hindering the acceptance to follow the pharmacological recommendation, to perform glycemic control, as well as to adopt a healthy diet and practice regular physical activities^(10-11)^. Consequently, this refusal can directly interfere with the patient’s prognosis.

Attitude is a cognitive manifestation that influences decision making when facing an event^(12)^ and is extremely important in a health problem, because when a person understands and accepts his treatment, it is easier to face the disease and maintain well-being^(13)^. Therefore, attitude is a determining factor in the adoption of self-care measures^(14)^. Studies have highlighted that elderly people with diabetes who have a negative attitude show difficulty in managing their health-disease process and a greater chance of developing complications^(14-15)^.

The willingness to self-care allows the person to perform routine activities favorable to their well-being and coexistence in society. In situations of chronic disease, such as diabetes, its adoption is essential to prevent complications and avoid a worsening in the general health status^(16)^.

From this perspective, it is essential that nursing care, when attending elderly with diabetes, identify depressive symptoms and negative attitude towards self-care^(17)^ early since its aim is to promote independence and autonomy to the patient^(8)^. Such promotion can occur by raising awareness through health education, providing guidance for the adoption of a new lifestyle habit, which includes actions such as following a diet plan, monitoring blood glucose, performing regular physical activities, correctly using medication, and practicing foot care^(8)^.

Thus, the correlation of symptoms of depression, attitude, and self-care in elderly people with type 2 diabetes highlights the importance of multidimensional care and allows assisting in the implementation of interventions that can prevent the negative impacts on self-management.

## OBJECTIVES

To correlate depressive symptoms, attitude, and self-care of elderly people with diabetes mellitus.

## METHODS

### Ethical aspects

The project of this study was approved by the Research Ethics Committee, being developed according to the recommendations of Resolution No. 466/2012 of the National Health Council. At that moment, the participants signed the Informed Consent Form.

### Study design, period, and location

This is an exploratory, descriptive, and cross-sectional study, of a quantitative approach, and guided by the STROBE tool. It was developed within the Family Health Units linked to the Health Districts of the municipality of João Pessoa, state of Paraíba (PB). The municipality is divided into five Health Districts under the supervision of the Municipal Health Secretariat and has as its main objective the management and monitoring of the family health teams; to assure access to basic services to the population, specialized in hospital care in a comprehensive way, according to public policies.

The data collection period occurred from June to October 2019. Data were collected through individual interviews.

### Population and sample, inclusion and exclusion criteria

Sample selection was based on the proportional allocation of the number of elderly people with diabetes served by district, considering the fixed selection cost for all elements of the target population. Thus, the sample size obtained by the stratification procedure, considering a simple random sampling plan in each district, was: District I = 37; District II = 27; District III = 38; District IV = 22; District V = 18; Total = 142. A maximum of two patients per unit was established to operationalize data collection. One additional interview was performed in District I (n = 38) and II (n = 28), totaling 144 participants. Thus, for the composition of the sample, 72 PHC services were visited.

The inclusion criteria established for the participants were: being 60 years of age or older, having a medical diagnosis of type 2 diabetes, and being registered in the Family Health Unit. The exclusion criteria were: elderly people incapable of verbal communication and who do not have cognitive ability to answer the questions, assessed by means of the Mini Mental State Examination^(18)^.

### Study protocol

To obtain data regarding the sociodemographic profile, a semi-structured instrument was used; this instrument was submitted to the prior evaluation of judges who were masters and doctors in the area. It presents information about the Family Health Unit, the Family Health Strategy, and the following variables: neighborhood, sex, age group, marital status, occupation/profession, education, race, family income, and religion. The presence of depressive symptoms was assessed by applying the Geriatric Depression Scale (GDS) - translated and validated in Brazil. It is one of the most used instruments for the investigation of depressive symptoms in the elderly population. It is a reduced version of the original scale^(19)^, based on the items that most strongly correlated with the diagnosis of depression. Its score ranges from 0 to 15 points and contemplates the following cutoff points: less than or equal to 5 points means a normal individual or no depressive symptoms; above 5 points, an individual with depressive symptoms^(20)^.

The psychological attitude was assessed by the *Questionário de Atitudes Psicológicas do Diabetes* (ATT-19) [Psychological Attitudes of Diabetes Questionnaire], which evaluates the psychological adjustment to diabetes, developed as a response to the need to evaluate psychological and emotional aspects about the disease. Validated in Brazil in 2005, it is composed of 19 items, which include six factors: stress associated with diabetes, receptivity to treatment, confidence in treatment, personal efficacy, perception about health, and social acceptance. Questions 11, 15, and 18 have a reverse score. Each answer is measured by a five-point Likert scale. The total score value ranges from 19 to 95 points, and a score greater than 70 points indicates a positive attitude about the disease^(21)^.

Self-care was assessed by means of the Diabetes Self-Care Activities Questionnaire (DSCA), a version translated, adapted, and validated for the Brazilian culture. It has 15 items distributed in seven dimensions: General diet (two items); Specific diet (three items); Physical activity (two items); Blood glucose monitoring (two items); Foot care (three items); Use of medication (three items, used according to the drug regimen). It also includes other three items for the evaluation of smoking habits^(22)^.

It allows the assessment of a given behavior during the span of seven days, so the scores of each item can range from 0 to 7, and higher scores indicate better results. The items of the “Specific diet” dimension should be inverted as suggested in the revised version^(22)^: 7 = 0; 6 = 1; 5 = 2; 4 = 3; 3 = 4; 2 = 5; 1 = 6; 0 = 7.

### Analysis of results and statistics

The data obtained were stored in a Microsoft Excel spreadsheet. Double data entry was performed to ensure reliability in data compilation. Then, they were organized, coded, imported, and processed by the software Statistical Package for the Social Sciences for Windows, version 22.0, and descriptive and inferential analyses were performed. The dependent variable included in the study was “self-care” with diabetes; and the independent variables were “depressive symptoms” and “attitude”.

To verify the normality of the numerical data, the Kolmogorov-Smirnov test was used. The association among the variables occurred by means of the chi-square, Mann-Whitney, and Kruskal-Wallis tests. Spearman’s Correlation coefficient was used to correlate the scales. The significance level used for the statistical analyses was 5% (p ≤ 0.05). The reliability of the factors was evaluated by estimating the internal consistency through Cronbach’s alpha coefficient, with values between 0.70 and 0.99 being admitted as indicative of a reliable instrument^(23)^.

## RESULTS

In this study, we observed a higher frequency of females (66.7%), between 60 and 69 years (56.9%), with a partner (54.9%), 9 to 12 years of education (33.3%), family income between one and three minimum wage salaries (88.9%), and retirees (75%). In screening for symptoms of depression, 24.3% of the elderly showed depressive symptoms (Table 1).

**Table 1:** Distribution of the presence of symptoms of depression in elderly people with diabetes mellitus, João Pessoa, Paraíba, Brazil, 2019 (N = 144)

Symptoms of depression	n	%
Without symptoms	109	75.7
With symptoms	35	24.3
Total	144	100.0

As for the attitude of coping with the disease, 93.8% presented negative attitudes in coping with diabetes mellitus (Table 2).

**Table 2:** Distribution of attitudes towards coping with the disease of elderly with diabetes mellitus, João Pessoa, Paraíba, Brazil, 2019 (N = 144)

Attitude of coping with the disease	n	%
Negative	135	93.8
Positive	9	6.2
Total	144	100.0

Regarding self-care activities, higher adherence was observed in the activities: Taking insulin injections as recommended (6.7); Taking diabetes medications as recommended (6.5); Taking the indicated number of diabetes pills (6.5); and Following a healthy diet (4.8). In contrast, the activities that exhibited lower adherence were: Eating sweets (6.0); Checking blood sugar in the recommended number of times (2.8); and Performing specific physical activities (1.5) (Table 3).

**Table 3:** Distribution of self-care activities of elderly with diabetes mellitus, João Pessoa, Paraíba, Brazil, 2019 (N = 144)

Self-care activities	Adherence Mean (Standard-deviation)
Taking insulin injections as recommended	6.7 (0.74)
Taking diabetes medications as recommended	6.5 (1.39)
Taking the indicated number of diabetes pills	6.5 (1.40)
Eating Sweets	6.0 (1.74)
Following a healthy diet	4.8 (2.04)
Eating five or more servings of fruits and/or vegetables	4.7 (2.24)
Checking the inside of your shoes before putting them on	4.7 (2.95)
Examining your feet	4.5 (2.93)
Drying the spaces between toes after washing	4.5 (2.97)
Following dietary orientations	4.1 (2.20)
Exercising for at least 30 minutes	3.6 (2.72)
Checking your blood sugar	3.4 (2.76)
Eating red meat and/or whole milk products	3.3 (2.24)
Checking blood sugar the recommended number of times	2.8 (2.89)
Performing specific physical activities (walking, swimming, etc.)	1.5 (2.18)

* *SD - Standard-deviation.*

In the correlation between depressive symptoms, the self-care dimensions, and attitude, a negative and inversely proportional relationship was observed regarding “Physical activity” (p = 0.010) and “Foot care” (p = 0.006), showing that the increase in symptoms of depression can cause a decrease in self-care in these dimensions. The correlation between self-care and attitude showed a negative inverse proportional relationship with statistical significance between attitude and the “Foot care” (p = 0.009) dimension, showing that the decrease of foot care can be correlated to an increase of negative attitude (Table 4).

**Table 4:** Correlation between the dimensions of self-care with symptoms of depression and attitude of elderly with diabetes mellitus, João Pessoa, Paraíba, Brazil, 2019 (N = 144)

Self-care	Symptoms of depression		Attitude	
	r	*p* value^*^	r	*p* value^*^
General diet	-0.043	0.609	-0.120	0.150
Specific diet	-0.123	0.142	-0.138	0.098
Physical activity	-0.214	0.010	-0.086	0.304
Blood glucose monitoring	-0.201	0.247	-0.033	0.850
Foot care	-0.228	0.006	-0.218	0.009
Use of medication	0.062	0.788	0.163	0.480

* *Spearman's Correlation Test.*

## DISCUSSION

Regarding the sociodemographic characteristics, there was a higher frequency of females. This result confirms the general representation of Brazil’s elderly, mostly female, which is related to greater health care, lower exposure to violence and risk factors, as well as the increase in life expectancy of the female population^(3)^.

The sample consisted largely of elderly people up to 69 years of age. A study conducted in China with 108 elderly people with diabetes showed that age was negatively associated with knowledge about the disease, i.e., the younger the age, the higher the level of knowledge^(24)^. This was similarly found in a randomized clinical trial with elderly diabetics in Primary Health Care in Recife, Northeast Brazil, where aging was associated with declining cognitive and motor skills and increased need for support for diabetes self-care management^(14)^.

A stable union was mentioned in most situations, corroborating a survey conducted with 301 people over 60 years linked to the *Núcleo de Atenção ao Idoso* [Center for Elderly Care] of the *Universidade Federal de Pernambuco*, in which it was found that the largest number of interviewees were married^(25)^. A study conducted in Paraná with 30 participants who lived with a spouse concluded that diabetes care among elderly couples involves actions such as checking blood glucose levels, taking medications, and preparing healthy food, which requires discipline and attention because they are activities that need to be performed every day. Moreover, it was observed that family support favors adherence to treatment and contributes to the prevention of complications^(26)^.

Most participants in this study did not have more than 12 years of education. In a study conducted in Rio Grande do Sul with elderly people with diabetes monitored in the Family Health Unit, the low level of education was related to the difficulty in managing self-care, due to the lack of understanding of therapeutic behaviors^(12)^. This same relationship was also reported in the *Centro Estadual de Atenção Especializada* [State Center for Specialized Care] of Viçosa, Minas Gerais, where patients with low education showed little or no willingness to deal with the disease^(27)^.

As for income, retirement was predominant in the study’s population, either by years of service or pension, which eventually interferes with access to health services and may be insufficient to meet basic needs, reducing adherence to treatment^(3)^.

In elderly people with diabetes, cognitive deterioration and physical disability are considered a major challenge to cope with the disease^(24)^; in addition, they are more vulnerable to the development of mental disorders such as depression^(9)^. For this reason, both the American Geriatrics Society and the American Diabetes Association recommend an active search for depressive symptoms during the first consultation and follow-up of these patients^(28)^.

The present study showed that a small portion of the interviewees had symptoms of depression, which can be justified by the follow-up performed in the Family Health Strategy, where the patient continuously attends nursing consultation^(13)^. Other studies have highlighted that elderly people with diabetes are less likely to develop symptoms of depression when receiving nursing care in Primary Care^(14-15)^.

The nursing care plan for the elderly person with DM includes interventions based on encouraging the patient’s participation in health education activities, facilitating the involvement in groups for the elderly, counseling for changes in lifestyle and adoption of healthy habits, as well as talking about the importance of drug therapy, clarifying doubts, in addition to listening and understanding complaints^(4-7)^.

In the results of a review, a prevalence of 10.6% for the most severe symptoms of depression, 17% for moderate to severe symptoms, and 11.6% for mild symptoms was found in elderly people with diabetes^(29)^. A study conducted in Ribeirão Preto with 121 elderly individuals at the Diabetes Outpatient Clinic of a general hospital showed that patients with depressive symptoms presented low average levels of regular physical activity and healthy diet^(5)^. The importance of the assistance provided to these patients in the Family Health Strategy is evident, where it is possible to use strategies to involve the elderly in the management of their health condition through nursing consultations, health education, and elderly groups, among others^(13)^.

The elderly assessed in this study showed a negative attitude towards diabetes. A similar result was identified in a randomized clinical trial that included 218 elderly people with diabetes treated at the Family Health Units in Recife - PE, where the same questionnaire of psychological attitudes towards diabetes was used. However, the researchers, through a problematizing group intervention, managed to influence the attitude of coping with the disease, thus increasing adherence to physical activity and a healthy diet and favoring the reduction of overweight and glucose adequacy of the participants^(14)^.

The absence of a positive behavioral attitude to deal with diabetes treatment for a long period of time can silently cause the dysfunction and failure of several organs, with consequences such as blindness, kidney failure, and lower limb amputations, generating significant health costs and restricting the ability to work as well as life expectancy^(15)^.

In a randomized clinical trial with 197 people over 40 years of age with diabetes in a Family Health Unit in the South Region of Brazil, an increase in positive attitude to face the disease was noted, which in turn favored the practice of self-care after educational interventions during nursing consultations^(30)^.

In this study, the greater adherence to self-care activities related to medication is similar to the data found in a cross-sectional study conducted in São Paulo - SP, which showed that participants had good acceptance for drug treatment, but low adherence to the diet plan, glycemic monitoring, and physical activity^(31)^. This finding may be related to the belief that medication produces better results in glycemic control than diet and physical exercise, so they adhere more to the use of drugs than to changes in lifestyle habits^(31)^.

Blood glucose monitoring showed low adherence. This may be associated with the fact that its realization depends on the acceptance of a healthy lifestyle, but changing habits is one of the biggest challenges for the treatment of diabetes, as the adaptation depends on factors related to psychological and socioeconomic conditions, as well as cognitive and health^(8)^. It is noteworthy that it is through nursing care that the related problems are identified; and, based on this information, the planning and implementation of interventions are performed^(6)^.

Participants in this study presented a high consumption of sweets, complicating glycemic control. It is assumed that non-adherence to this practice may be related to the difficulty in changing lifestyle habits, which significantly interferes with individual and family routine^(30)^. Something similar happens with the performance of physical activities since most interviewees do not practice them. Among the causes that prevent the adoption of regular physical exercise are socioeconomic problems, indisposition, unavailability, and lack of knowledge of the benefits^(32)^.

The Brazilian Diabetes Society^(3)^ refers to physical activity as one of the pillars of the disease treatment, since its regular practice of at least three times a week, preferably under professional guidance, is associated with reduced body weight, improved muscle tone, heart rate, and respiratory function. Furthermore, it favors the control of glycemic levels and decreases the risk of coronary diseases^(32)^.

In the correlation of depressive symptoms with the dimensions “Physical activity” and “Foot care”, a negative and inversely proportional relationship was observed, evidencing that the increase in depression symptoms can cause a decrease in self-care. The same occurred in the correlation between self-care and coping attitude.

The attitude towards diabetes was evaluated in China, in eleven thousand participants over 40 years of age; and a low score of coping was evidenced in patients who did not practice physical activity and in those who underwent psychological monitoring to deal with depression^(24)^. The literature points out that symptoms of depression in elderly people with diabetes are often related to the difficulty in performing self-care, especially in activities that require greater attention, such as foot care^(29)^.

Negative attitudes result from emotional demotivation with the health-illness process, reducing patients’ involvement in their treatment. This may justify the findings of this study, since foot care implies changes in routine, time, and discipline, as it requires daily inspection^(8)^.

The evolution of diabetic foot complications includes the occurrence of infection, possible ulcers, and amputations, with negative repercussions on disability. Therefore, the information offered by nurses is essential for patient awareness and refers to guidelines on hygiene, nail cutting, callus removal and skin hydration, as well as the use of appropriate footwear and daily physical examination of the feet^(30)^.

In this context, nurses should develop actions for the prevention of depressive symptoms, positively boosting the attitude of the elderly towards treatment, stimulating self-care measures. Among the approaches that can be performed are the use of measurement scales and screening for the presence of depressive symptoms, monitoring, and individualized consultations with the patient, groups of elderly with diabetes, and multidisciplinary interaction with the health team^(6)^.

### Study limitations

The limitation of this study refers to its cross-sectional design, which does not allow establishing a cause-and-effect relationship. Thus, longitudinal research is recommended to enable a long-term study and a comprehensive evaluation of the impact of depressive symptoms on attitude and self-care in this population.

### Contributions to the field of Nursing, Health, or Public Policies

This research provides evidence that can support the multidisciplinary health care of elderly people with diabetes mellitus, especially nurses who provide continuous and comprehensive care. It provides support in the development of a care plan directed to the needs of this clientele, with emphasis on the early identification of depressive symptoms and specific interventions in a timely manner, aiming to minimize their impact on the attitude to face the disease and on the maintenance of self-care activities. In addition, this study contributes to the advancement of research related to this specific population, bringing innovations by verifying the existence of correlations between depressive symptoms, attitude, and self-care.

## CONCLUSIONS

The results showed that 24.3% of the sample had depressive symptoms and almost all participants showed a negative attitude towards coping with the disease. The evaluation of self-care showed greater adherence to the activities of taking insulin injections and medications as recommended, taking the indicated number of diabetes pills, and following a healthy diet. On the other hand, the activities that exhibited lower adherence were: eating sweets, checking blood sugar the required number of times, and performing specific physical activities.

In the correlation between the scales of depressive symptoms and self-care, there was a negative and inversely proportional correlation between depressive symptoms and the dimensions “Physical activity” and “Foot care”. The same occurred with the correlation of self-care with the coping attitude, showing that self-care is influenced by depressive symptoms and negative coping attitude and can be impaired by them.
